# Twelve-Year-Old Girl with Primary Biliary Cirrhosis

**DOI:** 10.1155/2012/937150

**Published:** 2012-11-27

**Authors:** Ivana Kitic, Aleksandra Boskovic, Ivica Stankovic, Dragan Prokic

**Affiliations:** Department of Gastroenterology and Hepatology, Mother and Child Health Care Institute, Radoja Dakica 6-8, Belgrade, 11070 New Belgrade, Serbia

## Abstract

Primary biliary cirrhosis (PBC) is a slowly progressive cholestatic liver disease of autoimmune etiology. The initial presentation of PBC is varies from asymptomatic, abnormal liver biochemical tests to overt cirrhosis. Unlike other autoimmune liver diseases, PBC has rarely been reported in childhood. We report a case of primary biliary cirrhosis in a 12-year-old girl. In addition to characteristic histology features, strongly positive antimitochondrial antibodies, increased liver enzyme levels, increased serum quantitative immunoglobulin M levels, and cholestasis were discovered. She had been treated with ursodeoxycholic acid. In the world literature, we found only few pediatric patients of primary biliary cirrhosis. Aetiology, pathogenesis, the long-term natural history, and prognosis remain obscure. Due to increased awareness of early-onset PBC, rather than typical older ones, further pediatric cases may be discovered.

## 1. Introduction

Primary biliary cirrhosis (PBC) is a chronic, autoimmune (strongly associated with positive antimitochondrial antibodies AMA), progressive liver disease of unknown aetiology, that selectively affects the small intrahepatic bile ducts, characterized by their granulomatous destruction, portal inflammation, and scarring [1–3]. Cirrhosis appears only late in course. Pathogenesis of PBC is still unknown. Auto-aggression originates chronic inflammatory process through molecular mimicry caused by an external antigen [3]. Prevalence of PBC ranges 25–50/100000 inhabitants, the incidence is 2,7–3,5 cases per 100000 inhabitants/year [3]. Unlike other autoimmune liver diseases, PBC has rarely been reported in childhood [4]. In available literature, we found only few reports [5–7]. We present a case of primary biliary cirrhosis in a 12-year-old girl. 

## 2. Case Report

Ten days prior to presentation, she was examined, at secondary clinical center, for fever without an obvious source and cured with Amoxicillin. She also reported fatigue, weakness, right upper quadrant pain without weight loss. 

She was presented to our hospital (tertiary clinical center) with jaundice. Physical examination revealed enlarged liver and spleen. She was afebrile with normal blood pressure, regular heart, and respiratory rate.

**Figure 1:**
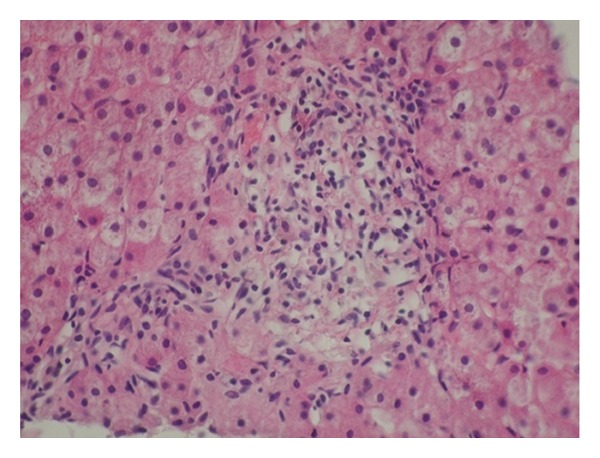
A portal tract shows a dense infiltrate of lymphoid cells and bile ducts disappeared leaving a granulomatous focus. The finding is consistent with stage I PBC (HE, 400x).

**Figure 2:**
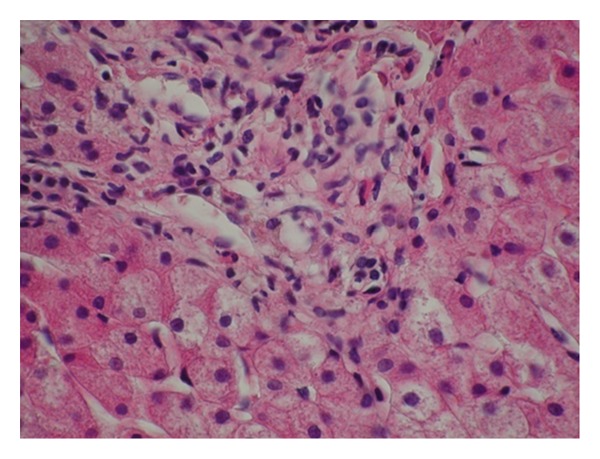
Granulomatous destruction of interlobular bile ducts with epitheloid histiocytes and eosinophils (HE, 600x).

Initial laboratory examination showed normal complete blood count, elevated alanine aminotransferase 680 IU/L (normal 5–40 IU/L), aspartate aminotransferase 343 IU/L (normal 5–40 IU/L), alkaline phosphatase 686 IU/l (normal < 332 IU/L), gamaglutamyl-transpeptidase 20 IU/L (normal < 20 IU/L), total bilirubin of 111,3 micromole/L (normal < 20 micromole/L) direct bilirubin of 48,4 micromole/L, and IgM 2,29 g/L level (normal 0,63 ± 0,3 g/L). Prothrombin time (PT) and the partial thromboplastin time (PTT) were normal. Serology for viral hepatitis A, E, B, C, and Epstein-Barr was negative. Abdominal ultrasonography, computed tomography, and magnetic resonance cholangiopancreatography did not show compression, obstruction, or hepatobiliary anomalies. Other causes of chronic cholestasis were considered but Alpha_1_-antitrypsin and ceruloplasmin serum levels were normal. We performed a percutaneous liver biopsy two months later because of chronic cholestasis. Serial cuts were stained by conventional histologic techniques (HE, PAS, PAS-D, Masson trichrome, reticulin, and tested for iron and copper). Microscopically all visible portal tracts exhibit dense infiltrate of small cells, histiocytes, and a few eosinophils. There was random, focal destruction of interlobular and septal bile ducts by early granulomatous inflammation (florid duct lesion) (Figures 1 and 2). Giant cells were not found. Changes of the lobular parenchyma were minimal including Kupffer-cell hyperplasia (with deposition of PAS positive material). Necroinflammatory process was limited to portal tract. Interface activity was not found like initial fibrosis as well. Focal central cholestasis was present. The liver biopsy showed stage I of PBC. Additional investigation showed positive AMA-M2 in high titter (first 1 : 80), and second after three months (1 : 300), negative antinuclear antibodies (ANA), smooth muscle antibodies (SMA), liver-kidney-microsome antibodies (LKM), and antibodies against cytoplasm antigens of neutrophiles (ANCA) and supported evidence for diagnosis of PBC. 

We started treatment with ursodeoxycholic acid 15 mg/kg/day. After three months of treatment, her liver enzymes were almost normal. In seven years time, no signs of disease reactivation have been found but AMA were still positive. Rebiopsy was not preformed because parents do not allowed.

## 3. Discussion

The term primary biliary cirrhosis (PBC) was established by Ahrens et al. in 1950 [8]. Primary biliary cirrhosis predominantly affects middle aged women (in 90% of patients) [1, 2]. Almost 60% of PBC patients are asymptomatic and commonly incidentally diagnosed. Asymptomatic stage lasts from ten to twenty years [1]. Actually, in 5% of AMA-positive patients asymptomatic stage lasted longer than twenty years. Symptomatic stage of PBC occurs as oligosymptomatic, symptomatic anicteric, icteric, and final (cirrhotic) stage. Fatigue, pruritus, jaundice, right upper quadrant pain, hepatosplenomegaly, hyperpigmentation, steatorrhea, osteoporosis occur in various liver diseases as in PBC. Dividing in precirrhotic and cirrhotic stage is important for clinical practice. Common extrahepatic manifestations of PBC include thyroid disease, CREST syndrome, scleroderma, Sjögren's syndrome, systemic lupus erythematosus, rheumatoid arthritis, polymyositis, glomerulonephritis, and renal tubular acidosis. However, celiac disease, hemolytic anemia, inflammatory bowel disease are uncommon extra-hepatic manifestations of PBC. Antimitochondrial antibodies (titers > 1 : 40), the serologic hallmark of PBC, are rarely associated with any other clinical condition [2]. Several AMA subtypes (M1, M3, M5, M6) are seen in patients with syphilis, pseudo lupus, collagenoses, and drug-induced liver disease. Along with high specific and sensitive AMA, other laboratory features of PBC include elevation of serum alkaline phosphatase, elevation of serum gamaglutamyl-transpeptidase, and elevated immunoglobulin M [2]. Ultrasonography, computed tomography, and magnetic resonance cholangiopancreatography are used for differential diagnosis. Liver biopsy is not necessary for all patients with PBC [2]. Histomorphologic pattern of PBC is characterized by granulomatous destruction of bile ducts, portal/periportal inflammation, and scarring. In 1965, Rubin et al. proposed the morphologic changes of PBC into four successive stages: portal stage, periportal stage, septal fibrosis, and cirrhosis [9], a staging system which has been promoted by other authors (Scheuer 1967, Popper and Schaffner 1970, Ludwig et al. 1978) [10–12]. The value of staging needle biopsy is conceptually useful but PBC is focal and variable disease and exhibits different degrees of severity in different portions of the liver. Occasionally all four stages may be found in a single liver removed at transplantation. Epitheloid cell granulomas, particularly when they are intimately associated with damaged bile ducts are a characteristic pathohistologic finding of PBC [13]. 

When diagnosis is established, therapy must be considered. Until now, there was no causal and specific treatment for PBC. Ursodeoxycholic acid (UDCA) is approved by Food and Drug Administration (FDA) for patients with PBC with adequate dosing—15 mg/kgBW/day [14]. In advanced stages, some of treatment combinations are reasonable (UDCA + prednisolon, UDCA + azathioprin) [14].

We presented a 12-year-old girl patient with primary biliary cirrhosis. This patient's characteristic histology feature, positive antimitochondrial antibodies (AMA), increased liver enzyme levels, and increased serum quantitative immunoglobulin M levels supported the diagnosis of PBC. We reported a well-documented case of pediatric onset PBC with followup of 5 years since diagnosis, respectively. We also found few reports about pediatric onset of primary biliary cirrhosis. Authors described two girls with PBC-aged 15 and 16 years with followup of 7 and 4 years since diagnosis [5]. Invernizzi et al. described a case of type 2 AIH with serological positivity for PBC-specific antimitochondrial antibodies (AMA) in a 3-year-old girl [6]. Melegh et al. described a newborn girl with a history of connatal liver damage and very high titres of antimitochondrial antibodies that were later detected in the plasma. As the hepatic injury tended towards fibrosis, the histological diagnosis, at the age of six, became primary biliary cirrhosis [7]. We reported the first case of PBC in previously healthy young child. 

Aetiology, pathogenesis, the long-term natural history, and prognosis remain obscure. The real incidence and natural history of PBC in childhood is unknown. Due to increased awareness of early-onset PBC, rather than typical older ones, further pediatric cases may be discovered.
